# The Agro-Physiological and Phytochemical Potential of Quinoa (*Chenopodium quinoa* Willd.) Under Saline Stress: A Comprehensive Investigation of Nutritional Properties and Antioxidant Activities

**DOI:** 10.3390/plants14101482

**Published:** 2025-05-15

**Authors:** Marwa Zouari, Wafa Rjeibi, Mohamed Hachicha

**Affiliations:** Laboratory of Valorization of Non-Conventional Water, National Research Institute of Rural Engineering, Water and Forestery, University of Carthge, 17 Hedi Karray route, 2080 Ariana, Tunisia; wafarjeibi2@gmail.com (W.R.); mohamed.hachicha@ingref.ucar.tn (M.H.)

**Keywords:** quinoa, saline water, crop physiology, biochemical parameters, nutritional composition, mineral elements

## Abstract

This study was conducted to evaluate the effects of varying irrigation salinity levels on the physiological and biochemical responses of Chilean white quinoa (*Chenopodium quinoa* Willd.) in two greenhouse cultivation seasons (2015 and 2016) in Cherfech, Tunisia. The experiment involved three treatments: T0 (control, using tap water with EC = 1.4 dS/m), T1 (EC = 9.0 dS/m), and T2 (EC = 18 dS/m). The results showed significant differences with multiple parameters. Quinoa irrigated with T2 exhibited superior growth and seed yield compared to the other treatments in both seasons. Additionally, soluble protein content increased significantly in the second season, especially in T2, while nitrogen content also rose notably from T0 to T2 in 2016. Bioactive compounds, such as vitamin C, phenols, flavonoids, DPPH, and ABTS, were highest in T2. Mineral content (K, P, Na, Mg, Zn, Ca, and Fe) showed considerable variation, with T2 generally presenting the highest levels. Amino acids exhibited notable changes, with an increase from T1 to T2, though lysine content decreased under T2 conditions. These findings indicate that quinoa can effectively adapt to saline irrigation, positioning it as a promising crop for cultivation in saline-affected regions.

## 1. Introduction

Tunisia, as a Mediterranean country, faces critical challenges in ensuring agricultural productivity due to its predominantly arid and semi-arid climate. The increasing scarcity of water resources and the prevalence of salt-affected soils significantly constrain agricultural practices and food security [1]. Over 20% of irrigated lands and 6% of global land areas are affected by salinity, a percentage projected to rise due to climate change and unsustainable agricultural practices (FAO, 2021). Soil salinization not only impacts crop productivity but also exacerbates the existing food security crisis, particularly in regions like Tunisia where cereal production is insufficient to meet local demand. Developing innovative strategies to enhance crop resilience under such conditions has become imperative, and quinoa presents a promising solution due to its superior salt tolerance and economic potential.

Currently, global concerns about soil salinity are intensifying due to its widespread impact on agricultural productivity and its exacerbation by climate change. These challenges are compounded by the growing demand for food, driven by an expected population increase to 9 billion by 2050 (UN, 2022). Addressing these issues requires resilient crops capable of thriving under abiotic stress conditions, particularly salinity (FAO, 2021).

Among the proposed solutions, *Chenopodium quinoa* Willd. has emerged as a promising crop for addressing these challenges, not only due to its exceptional ability to withstand high salt levels but also because of its remarkable nutritional profile.

Quinoa, a pseudocereal native to the Andean region, is recognized for its adaptability to adverse environmental conditions, including drought and salinity [2]. It exhibits high salt tolerance, capable of withstanding salinity levels up to 750 mM NaCl, surpassing staple crops such as barley, wheat, and maize [3]. Quinoa’s tolerance mechanisms include osmotic stress mitigation, ion regulation, and antioxidant defenses, such as enhanced activities of catalase, peroxidase, and ascorbate peroxidase enzymes [4]. This adaptability makes quinoa an ideal candidate for improving agricultural productivity in regions where salinity limits crop growth.

Recent studies have further highlighted quinoa’s potential under varying environmental conditions. Research on three accessions (18 GR, R-132, and DE-1) has revealed significant variability in their response to salt stress [5]. Greenhouse conditions were found to promote faster growth, higher photosynthetic activity, and increased enzymatic responses compared to field conditions. Specifically, the DE-1 accession demonstrated superior salt tolerance at 200 mM NaCl, maintaining higher photosynthetic rates and lower oxidative stress markers (e.g., MDA and H_2_O_2_ levels) under both conditions. This suggests that selecting and optimizing specific quinoa accessions, such as DE-1, can enhance productivity in saline-prone regions [6].

Similarly, an open-field study on two quinoa accessions (27 GR and Line 0291) revealed distinct responses to salinity [6]. The Line 0291 accession exhibited greater salt tolerance by accumulating osmolytes like proline and sugars, maintaining higher water content, and achieving better grain yield under salt stress compared to 27 GR. These findings underline the importance of osmotic adjustment mechanisms and suggest that Line 0291 has a higher capacity to sustain photosynthetic activity and productivity under saline conditions [6].

In addition to its resilience, quinoa’s nutritional attributes make it a valuable crop for addressing global food security challenges. It is rich in high-quality proteins, essential amino acids, and bioactive compounds, including phenolics and flavonoids, which contribute to its antioxidant properties. These traits enhance its potential as a functional food, particularly in regions where nutritional deficiencies prevail [7]. Despite its advantages, the use of quinoa in sustainable agricultural systems, especially in saline and arid environments like Tunisia, remains underexplored. Studies have shown that organic amendments, such as cotton shell biochar, can enhance plant salt tolerance by ameliorating Na-induced phytotoxicity and improving soil properties [8]. However, there is a pressing need for field-based investigations to confirm these findings and optimize quinoa cultivation in real-world conditions, specifically addressing the environmental and socio-economic conditions in Tunisia.

This study aims to evaluate the agro-physiological and phytochemical responses of quinoa to saline stress, focusing on growth, yield, and secondary metabolite production. By analyzing the effects of varying saline water concentrations on multiple quinoa accessions, this research seeks to identify mechanisms underlying salt tolerance and provide insights for integrating quinoa into Tunisia’s agricultural systems, particularly in saline and arid environments. Furthermore, it addresses the socio-economic implications of adopting quinoa as a crop to enhance food security and foster rural development.

## 2. Results

### 2.1. Soil Physicochemical Quality

The analysis of Table 1 reveals differences in the initial soil physicochemical conditions between the two quinoa growing seasons. In 2015, the soil pH was 7.9, while in 2016, it increased slightly to 8.1. The ECe rose from 2.63 dS/m in 2015 to 2.98 dS/m in 2016. While Ca^2+^ and Mg^2+^ concentrations remained stable, Na^+^ and Cl^−^ levels increased in 2016. This increase could be due to the higher soil pH in 2016, which may have enhanced the solubility and mobility of Na+ and Cl-, as more basic conditions favor salt dissolution. Additionally, the increased ECe indicate a buildup of salts, likely contributing to the higher Na^+^ and Cl^−^ concentrations. Nutrient concentrations, including nitrogen (N), phosphorus (P), and potassium (K), showed minimal variation, with N remaining stable at 0.8 mg/kg, P slightly increasing from 16.2 mg/kg to 16.5 mg/kg, and K rising from 60.2 mg/kg to 63 mg/kg. These results provide insights into the soil conditions, highlighting a basic pH, moderate salinity, and stable nutrient levels, which can influence plant growth and salt tolerance in quinoa.

### 2.2. Panicule Height and Quinoa Yield Components

**Figure 1:**
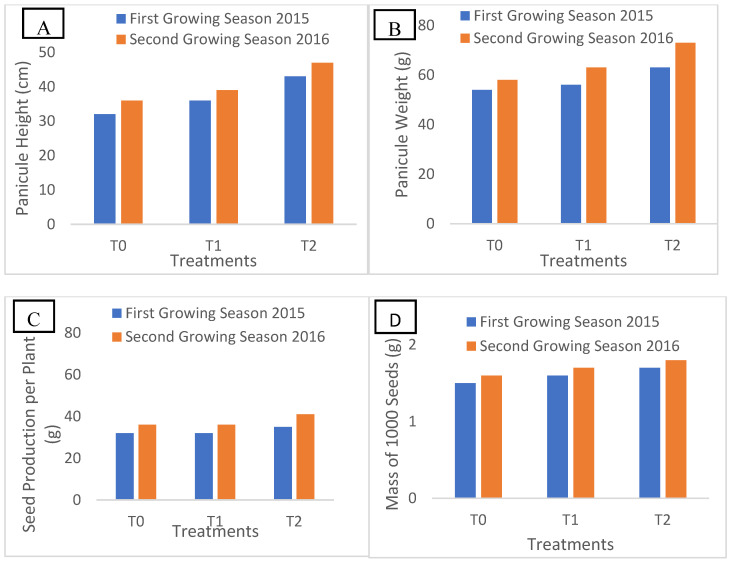
Panicule weight (g) (**A**), pinicule height (cm) (**B**), mass of 1000 seeds (g) (**C**), and seed production (g/plant) (**D**) during two growing seasons. Note: Means followed by different letters above the bars indicate significant differences (*p* < 0.05) between the different treatments: T0 (control, using tap water with EC = 1.4 dS/m), T1 (EC = 9.0 dS/m), and T2 (EC = 18 dS/m) according to the Tukey test.

Figure 1 presents the quinoa growth parameters at maturity over the two years of experimentation under three distinct treatments (T0, T1, and T2). These treatments were designed to represent a range of saline conditions typically encountered in Tunisian agricultural environments, with T0 as the control, T1 simulating moderate salinity, and T2 representing high salinity levels commonly found in regions impacted by saline water irrigation or coastal soil salinization These conditions are relevant for evaluating quinoa’s adaptability and resilience to the salinity stress typically observed in Tunisian agricultural regions.

In 2015, significant variations were observed in panicule height, panicule weight, seed production per plant, and 1000-seed mass among these groups, with maximum values recorded in treatment T2, followed by treatment T1, and minimum values in the control treatment. These findings indicate a positive correlation between saline irrigation and improved growth parameters, suggesting that moderate saline stress (T2) can enhance quinoa productivity. These results were also consistent in 2016, confirming the significant impact of irrigation water quality on quinoa growth parameters. These results over the two years suggest that quinoa can be cultivated under varying saline conditions without significant yield loss, making it a resilient crop for semi-arid regions.

### 2.3. Soluble Protein and Nitrogen Content of Quinoa Grains

**Figure 2:**
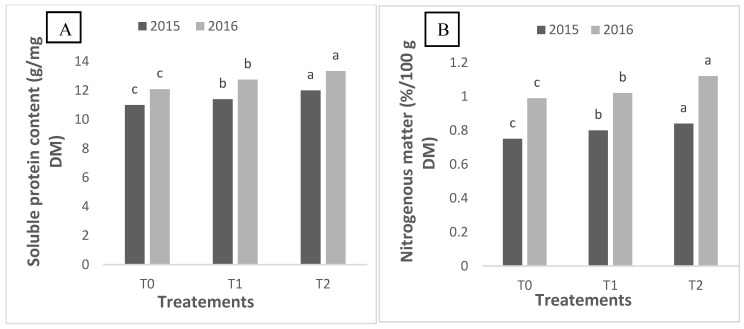
Soluble protein content (**A**) and nitrogen content percentage (**B**) of quinoa seeds grown in 2015 and 2016 and irrigated with different irrigation water treatments. Note: Means followed by different letters above the bars indicate significant differences (*p* < 0.05) between the different treatments: T0 (control, using tap water with EC = 1.4 dS/m), T1 (EC = 9.0 dS/m), and T2 (EC = 18 dS/m) according to the Tukey test.

The analysis of soluble protein content in white quinoa seeds at the end of the quinoa growing season revealed significant variations between treatments and years (Figure 2). An increase in soluble protein content was observed from year to year, indicating a positive response of quinoa to successive growing conditions (Figure 2A). In 2016, the treatment with the highest salt concentration, T2, showed a significantly higher soluble protein content compared to treatments with lower concentrations (T0 and T1). This result suggests that higher salinity may enhance quinoa’s ability to synthesize soluble proteins, possibly as a response to osmotic stress. This observation confirmed the positive impact of increasing salt concentration in irrigation water on soluble protein content. Interestingly, even in 2015, treatment T2 showed significantly higher soluble protein content, demonstrating that quinoa can adapt and maintain higher protein production under saline conditions.

Figure 2B shows the analysis of nitrogen content in white quinoa seeds at the end of the vegetative cycle, revealing significant differences between treatments. Nitrogen content significantly increased from T0 to T2. This suggests that quinoa may be more efficient in nitrogen uptake and assimilation under saline conditions. In 2016, similar variations were observed, but with an overall increase in nitrogen content in all treatments compared to the previous year. The increase in nitrogen content may indicate that quinoa is adapting to successive growing seasons by improving its nutrient uptake mechanisms in response to environmental stress.

### 2.4. Chemical Composition of Quinoa Seeds

The data in Table 2 show significant differences (*p* < 0.05) for all parameters analyzed in quinoa seeds irrigated with different saline water treatments in 2015 and 2016. In both years, treatment T2 (EC = 18 dS/m) consistently exhibited the highest values for vitamin C, total phenols, ortho-diphenols, flavonoids, ABTS, and DPPH, compared to T0 (control, EC = 1.4 dS/m) and T1 (EC = 9.0 dS/m). These results suggest that quinoa plants respond to increased salinity by producing higher levels of antioxidants and phenolic compounds, which likely serve as a defense mechanism against salt stress. Significant differences in the levels of these compounds were observed between the treatments, indicating that quinoa’s metabolic responses are significantly influenced by salinity.

Furthermore, the results from 2016 showed a trend of increased values for all parameters compared to 2015, likely due to the plant’s adaptation to the saline environment over time. This highlights the progressive acclimatization of quinoa to salinity stress.

The results are presented on a dry weight (DW) basis, with the following units: ascorbic acid (AA) for vitamin C, gallic acid equivalents (GAE) for total phenols and ortho-diphenols, catechin equivalents (CE) for flavonoids, and Trolox equivalent antioxidant capacity (TEAC) for ABTS and DPPH.

Each value is the mean ± standard deviation. The letters above the bars denote significant differences between treatments according to the Tukey test (*p* < 0.05).

### 2.5. Mineral Content

The analysis of the mineral element content in quinoa seeds in 2015 revealed significant variations between treatments (Table 3). For K, the concentration significantly increased from treatment T0 to treatment T2. Although the numerical differences between treatments were relatively small, statistical analysis confirmed their significance (*p* < 0.05), ensuring the reliability of the observed trends. This trend was consistent in 2016, with T2 exhibiting the highest concentration compared to T0 and T1.

For Na, a similar trend was observed, with significant increases between treatments in both growing seasons. The clustering of values, particularly for Na, was analyzed in detail, and the Tukey test confirmed that the observed differences between treatments were statistically significant. Regarding P, a marked and significant increase was recorded from T0 to T2, particularly in 2016, where T2 exhibited a 27% higher concentration compared to T0.

For Mg, the concentration significantly increased across treatments, with T2 consistently showing the highest values in both 2015 and 2016. The statistical analysis validated these differences despite the relatively close values between treatments. A similar trend was observed for Zn, with T2 presenting significantly higher concentrations than T0 and T1, supporting the role of salinity in enhancing zinc accumulation in quinoa seeds.

For Ca and Fe, significant variations were also observed. In both years, T2 exhibited higher concentrations compared to the control (T0), and statistical analysis confirmed the differences were significant (*p* < 0.05) across replicates.

### 2.6. Amino Acid Concentrations

Table 4 presents the amino acid concentrations in quinoa seeds for both growing seasons under different treatments (T0, T1, and T2). For the first growing season, significant differences were observed between treatments. The amino acid values, such as Trp, His, Thr, Val, Lys, Leu, Phe, Tyr, Asp + Asn, Ser, Glu + Gln, Gly, Arg, and Ala, showed a gradual increase from T1 to T2 compared to the control. However, it is important to note that Lys decreased from T1 to T2 compared to the control, suggesting a variable response of amino acids to different treatments. This variation indicates that while most amino acids increased with saline stress, Lys showed a decrease, possibly reflecting its sensitivity to saline conditions. Regarding the second growing season, similar to the first year, there was a progressive increase in amino acid values from T1 to T2 compared to the control. Again, it is essential to emphasize that despite this increasing trend, Lys decreased from T1 to T2 compared to the control. This suggests a consistent response of Lys to saline irrigation across the two growing seasons. Comparing the two years, there was a remarkable increase in amino acid content, except for Lys, which showed a decrease.

### 2.7. Multivariate Analysis: PCA and Heatmap

The Principal Component Analysis (PCA) reduced the dimensions of the studied parameters (33 variables) into five principal components. Among these, the principal components PC1 (F1) and PC2 (F2) accounted for 86.46% and 9.52%, respectively, of the total variance caused by the three treatments tested on quinoa over two growing seasons (Figure 2A). This high cumulative variance (95.98%) highlights the efficiency of PCA in summarizing the complexity of the dataset while preserving meaningful variability. However, the results should be carefully evaluated, as many values are closely clustered, raising questions about the statistical significance of the observed patterns.

The Biplot analysis (Figure 2A) revealed distinct variations among treatments T0, T1, and T2 across the two growing seasons (2015 and 2016). In 2015, factor F1 was predominant for the control (T0), whereas treatment T1 was primarily influenced by F2. Similarly, treatment T2 in 2015 was also dominated by F2. In 2016, the control (T0) remained under the influence of F1, but treatment T1 displayed a more balanced contribution of F1 and F2. For treatment T2, F1 became the dominant factor during the same season. These observations underline the dynamic and season-specific effects of the treatments on quinoa growth parameters, reflecting interactions between environmental factors and treatment applications. Nevertheless, due to the clustering of values, further statistical testing is needed to confirm the robustness of these findings.

The combination of Biplot analysis (Figure 2A) and Heatmap visualization (Figure 2B) provided comprehensive insights into the complex relationships within the dataset. In 2015, for treatment T0, the variable weight of 1000 seeds showed strong associations with parameters such as panicle height, seed yield, and vitamin C. These interconnections suggest a high degree of dependence among these traits, potentially driven by shared physiological mechanisms. Notably, the variable Lysine (Lys) displayed a significant correlation with panicle height, highlighting its relevance to plant growth processes under treatment T0 in 2015. Lysine, a critical amino acid for protein synthesis, plays a pivotal role in cellular growth and structural formation.

In 2016, for treatment T2, robust correlations emerged between panicle weight and other parameters, including panicle height, seed yield, and vitamin C. This intricate network of relationships suggests that changes in one parameter might trigger similar variations in others, underscoring their interconnected nature. Interestingly, the variable Tyrosine (Tyr) was strongly correlated with all observed parameters, pointing to its central role in the metabolic adaptation of quinoa under treatment T2.

The analysis of proteins, nitrogen, and mineral elements, which are critical for plant development, further revealed significant correlations. For instance, soluble protein content was positively associated with panicle height and vitamin C, suggesting a bidirectional influence. Similarly, % nitrogen content correlated with parameters like panicle height and seed yield, highlighting nitrogen’s key role in promoting growth and productivity.

Regarding mineral elements (K, Na, P, Mg, Zn, Ca, Fe), notable correlations were observed with other parameters. For example, zinc (Zn) was strongly linked to soluble protein content and vitamin C, underscoring its importance in enzymatic activity and stress tolerance. Salinity stress, a critical factor in the experiment, induced profound metabolic and physiological changes, altering the composition of metabolites such as proteins, nitrogen, and minerals. The observed correlations indicate that quinoa plants adjust their metabolic processes to maintain functionality and resilience under saline conditions.

## 3. Discussion

The physicochemical properties of soil exhibiting significant variations between the quinoa growing seasons of 2015 and 2016 provide critical insights into agronomic performance. The marginal increase in soil pH from 7.9 to 8.1 indicates a shift toward more alkaline conditions, which may hinder nutrient bioavailability essential for quinoa growth [9]. Moreover, the elevation in electrical conductivity from 2.63 to 2.98 dS/m signifies increased salinity, posing potential challenges to water uptake and physiological health, even in light of quinoa’s established salt tolerance [10]. Despite stable levels of calcium and magnesium, the rise in sodium and chloride concentrations raises concerns regarding salinity stress, which can adversely affect root functionality and nutrient absorption [11].

While salinity was the primary factor studied, other environmental factors, such as temperature fluctuations, could also have contributed to the observed differences. Temperature variations may impact enzymatic activity and photosynthetic efficiency, which are integral to growth and yield. Therefore, adopting strategic management practices to optimize these soil properties for quinoa cultivation is imperative in the face of escalating salinity.

In light of these soil challenges, quinoa, recognized for its halophytic characteristics, exhibited marked growth variations under saline conditions across treatments T1 and T2, with significant differences observed in panicle height, biomass, and seed yield. These findings underscore the critical role of irrigation water quality in determining growth parameters. Treatment T2 consistently yielded superior results, demonstrating quinoa’s remarkable adaptability to fluctuating salinity levels [12]. The plant’s physiological mechanisms, which facilitate growth maintenance under adverse conditions, position quinoa as a promising crop for cultivation in saline areas, thereby enhancing food security in such regions [3].

Stable calcium and magnesium levels contribute to quinoa’s adaptation under salinity stress by maintaining critical physiological functions, such as ion transport and photosynthesis [13]. These elements play roles in stabilizing cell membranes and facilitating continued energy production, enabling quinoa to better withstand salt stress. Calcium stabilizes cell membranes and helps regulate ion uptake, which is essential under salt stress. Magnesium, as a key component of chlorophyll, supports continued energy production through photosynthesis, even under less-than-ideal conditions [14]. These stable levels suggest that quinoa can better withstand salinity stress without facing major disruptions in nutrient uptake and metabolic processes [15].

As quinoa thrives under these conditions, the adaptability of this crop to saline environments is further evidenced by notable variations in soluble protein and nitrogen content [16]. These physiological and biochemical traits, such as antioxidant levels and nitrogen uptake, are valuable markers for breeding programs focused on salinity tolerance. These traits can be targeted to develop quinoa varieties optimized for saline environments. The observed increase in soluble protein levels from 2015 to 2016 reflects quinoa’s positive physiological response to salinity, corroborating findings that link salt stress to enhanced protein synthesis [17]. Elevated nitrogen levels in treatment T2 indicate that increased salinity may facilitate improved nutrient uptake [17]. The increased nitrogen uptake in T2 could be attributed to enhanced root development, which allows for a greater surface area for nutrient absorption. Additionally, the salt stress may activate certain transporters in quinoa roots that are involved in nitrogen assimilation, such as nitrate reductase, which plays a key role in nitrate uptake and conversion into usable forms for the plant [18]. Moreover, osmotic adjustments made by quinoa under saline conditions may improve nutrient uptake efficiency, compensating for the osmotic stress caused by high salinity. These physiological mechanisms likely contribute to the observed increase in nitrogen levels in treatment T2, supporting quinoa’s adaptive response to saline stress. These insights emphasize quinoa’s potential as a sustainable crop option in saline regions.

Consistent with these findings, the work of [19] demonstrates that quinoa’s metabolomic profile is profoundly affected by saline–alkali stress, with 232 metabolites significantly altered, including flavonoids, lipids, phenolic acids, and amino acids. These compounds, particularly flavonoids and phenolic acids, play pivotal roles in quinoa’s response to stress. However, saline–alkali conditions were found to decrease flavonoid and phenolic acid concentrations, suggesting a metabolic trade-off under stress conditions. These observations highlight the importance of further metabolomic studies to elucidate pathways that could be optimized for stress resilience.

In addition to protein and nitrogen enhancements, the application of saline irrigation water substantially enhanced the bioactive compound profile of quinoa seeds, particularly with respect to vitamin C and phenolic compounds, most notably in treatment T2. The increase in antioxidant levels suggests that quinoa possesses the ability to accumulate protective compounds in response to salt stress [20]. The increase in antioxidants observed is a combination of a stress response and broader metabolic adaptation. The sustained high levels of antioxidants, like vitamin C, suggest a metabolic shift to optimize stress tolerance. This capacity not only enhances quinoa’s nutritional value but also solidifies its classification as a superfood within saline environments, thereby contributing to sustainable food production initiatives [18].

Specifically, the observed increase in vitamin C levels under saline conditions may enhance antioxidant defense mechanisms, reducing oxidative stress on cellular structures. This mitigation of stress preserves essential metabolic functions, such as photosynthesis, which supports improved seed development and yield.

Moreover, the influence of saline irrigation on quinoa’s amino acid profile is notable, revealing significant increases in essential amino acids while concurrently exhibiting reductions in concentrations across treatments T1 and T2. This decrease may be attributed to metabolic resource allocation or competitive interactions among amino acids during synthesis [21]. Overall, the enhancement in amino acid levels suggests an adaptive response to saline conditions, likely linked to improved root development and nutrient absorption capabilities [22]. Further investigation into the biochemical pathways regulating amino acid synthesis under salt stress is vital for augmenting quinoa’s nutritional profile. The decrease in lysine levels could be part of a resource allocation strategy, where lysine biosynthesis is deprioritized in favor of other metabolic pathways critical for stress adaptation, such as the production of proline or secondary metabolites.

Regarding yield decline at higher salinity levels, while quinoa demonstrated considerable resilience, long-term exposure to higher salinity may eventually affect yield. Our study did not observe significant yield decline in treatment T2, but further research with progressively higher salinity conditions is essential to determine the point at which yield is negatively impacted. The decrease in lysine levels may reflect a metabolic trade-off, where lysine biosynthesis is deprioritized in favor of other pathways critical for stress adaptation, such as proline production or secondary metabolites. It is important to note that while quinoa has shown adaptability to higher salinity levels, continuous exposure to extreme salinity could lead to a decline in yield due to factors such as osmotic stress and ion toxicity [23].

In addition to the highlighted physiological and metabolic responses, soil microbiome interactions can play a pivotal role in quinoa’s adaptability to saline conditions. Recent studies have shown that plant–microbe symbiosis, including the presence of rhizobacteria, can enhance nutrient uptake, improve stress tolerance, and even mediate salt tolerance mechanisms [22].

Notably, ref. [21] demonstrated that certain quinoa genotypes exhibit superior antioxidant activity and nutrient accumulation under saline stress, with significant correlations observed between traits such as phenol content, anthocyanins, and Na^+^/K^+^ ratios [24]. Our findings align with these results, as increased antioxidant levels and stable Na^+^/K^+^ ratios in treatment T2 highlight the genetic potential of quinoa to thrive in saline environments.

The correlation between seed yield and vitamin C observed in the PCA and heat map can be explained by vitamin C’s critical role in reducing oxidative stress. By mitigating ROS-induced damage, vitamin C ensures the maintenance of essential physiological processes such as photosynthesis, nutrient transport, and energy metabolism, which directly contribute to seed development and yield.

These insights underscore the necessity of identifying high-performing genotypes to maximize yield and nutritional quality.

Finally, the findings from the Principal Component Analysis (PCA) underscore quinoa’s adaptive responses to saline treatments across the two growing seasons, with Principal Component 1 (PC1) accounting for a substantial portion of variance in physiological responses. The observed differentiation among treatments highlights quinoa’s capacity to modulate its growth strategies in response to environmental shifts [21]. Furthermore, the correlations identified between seed weight and various growth parameters indicate interdependent physiological relationships that enhance quinoa’s resilience under stress.

Regarding growth consistency across replicates, variations were observed, which could be attributed to environmental factors or genetic variability within the quinoa population. Although significant trends were evident in overall performance, growth patterns were not entirely uniform across replicates, underlining the complexity of quinoa’s response to saline stress and the influence of external factors. Fluctuations in temperature may impact enzymatic activity and photosynthetic efficiency, which are integral to growth and yield.

## 4. Materials and Methods

### 4.1. Materials

#### 4.1.1. Physicochemical Quality of Irrigation Water

Table 5 presents the physicochemical quality of the different irrigation waters used during the experiment. Significant variations were observed among the three treatments (T0, T1, and T2) in terms of the physicochemical quality of irrigation water. The increase in electrical conductivity (EC) of irrigation waters from 1.4 to 18.0 dS/m was also accompanied by variations in other parameters. For instance, concentrations of Na^+^, Cl^−^, and K^+^ showed an increase with the elevation of EC. Similarly, concentrations of HCO_3_^−^, SO_4_^2−^, Ca^2+^, and Mg^2+^ increased as EC increased.

#### 4.1.2. Plant Material

In our study, we examined a single origin of quinoa, “Chilean white quinoa”, grown under greenhouse conditions in a plot located at the Cherfech station in Tunisia, during two growing seasons in 2015 and 2016. The Cherfech station is situated in a semi-arid area with a Mediterranean climate, in northern Tunisia. White quinoa was chosen because it is a food crop gaining popularity due to its exceptional nutritional qualities and ability to thrive in challenging environmental conditions, including saline stress. This variety is known for its resilience and adaptability to various environmental stressors, making it a suitable candidate for this study. Furthermore, it has been widely cultivated and studied in similar agro-climatic zones, which reinforces its selection as a reliable representative for evaluating quinoa’s performance under saline stress.

#### 4.1.3. Climatic Data

The primary climatic data for the growing seasons of 2015 and 2016 were gathered from the Cherfech weather station (Table 6). In March, the first month of the quinoa vegetative cycle, the average precipitation in 2015 was two and a half times greater than in 2016, measuring 133.2 mm compared to 48.2 mm. The table also illustrates the variations in minimum and maximum temperatures, as well as average relative humidity, between the two years.

#### 4.1.4. Cultivation and Harvest Conditions

The seeds used in this study were obtained from the harvest of an experiment conducted in 2014, where the plants were irrigated with tap water. The saponin content of the samples was assessed using the semi-quantitative method outlined by [7]. To prepare the seeds, the seed coats were carefully removed by rinsing with distilled water, a process that was repeated several times. On March 5, 2015, the seeds were placed in trays filled with commercial potting soil and allowed to germinate for 30 days. Once the quinoa seedlings developed three leaves, they were transplanted into greenhouse plots, and the experimental design was a Randomized Complete Block Design (RCBD) with three replications (blocks). Each treatment group (T0, T1, and T2) was represented by two rows within each block, with six seedlings transplanted per row, resulting in a total of eighteen rows. The transplantation was carried out over one week, using the same amount of tap water for all seedlings. One week after transplantation, saline treatment commenced, involving different salt concentrations. Throughout the experiment, the plants were irrigated with a consistent volume of water using a drip irrigation system. The three treatment groups were as follows: T0 (control = 2 dS/m), T1 (9 dS/m), and T2 (18 dS/m). Irrigation occurred every 48 h during the wet period and every 24 h during the dry period to maintain constant soil moisture levels. Seven days prior to the end of each experimental vegetative cycle, the plants were exposed to tap water. Harvesting took place at the end of the cycle, specifically at the end of June, during which various parameters were evaluated.

### 4.2. Methods

#### 4.2.1. Physicochemical Properties of Irrigation Water

Irrigation water was regularly analyzed to evaluate its physicochemical quality. The pH and electrical conductivity of effluent samples were measured weekly using a pH meter (The WTW inoLab® pH 7110 Benchtop Meter is manufactured by WTW, a brand under Xylem Inc., and originates from Germany.) and a conductivity meter (The WTW inoLab^®^ Cond Level 2 is manufactured by WTW, a brand of Xylem Inc., in Germany.), respectively. Additionally, the soluble ions in the irrigation water samples were periodically analyzed. The quantification of Cl^−^ and HCO_3_^−^ ions was performed through titration with silver nitrate (AgNO_3_) solution and hydrochloric acid (HCl), respectively. SO_4_^2−^ ions were measured using the nephelometric method, while the concentrations of Ca^2+^ and Mg^2+^ ions were determined through complexometric titration. Na^+^ and K^+^ ions were analyzed using The Jenway PFP7 is a flame photometer manufactured by Jenway in the United Kingdom.

#### 4.2.2. Physicochemical Properties of Soil

Soil samples were randomly collected at various depths from the Cherfech experimental plot using a Dutch auger. After collection, the samples were air-dried and sieved through a 2 mm mesh before being transported to the laboratory for detailed physicochemical analysis. The soil pH was measured in a soil–water mixture consisting of 20 g of soil and 50 mL of distilled water, with readings taken after 4 h. The electrical conductivity of the saturated paste extract (EC_e_) was assessed using a conductivity meter (WTW InoLab Cond Level 2), The WTW InoLab Cond Level 2 is manufactured by WTW, based in Germany following the procedures outlined by the US Salinity Laboratory staff (1954). The concentrations of Cl^−^, SO_4_^2−^, HCO_3_^−^, Na^+^, Mg^2+^, Ca^2+^, and K^+^ were determined from the saturated paste extract using the same methods applied for water sample analyses.

#### 4.2.3. Quinoa Analysis

##### Post-Harvest Analyses of Quinoa Yield Components

During the quinoa plant harvest, random samples were collected from each treatment to measure panicle length, the number of panicles per plant, and the number of seeds per panicle. The panicles from these samples were sun-dried for three days, then threshed and winnowed by hand to determine the weight of 1000 seeds and the individual seed yield.

##### Mineral and Biochemical Analyses of Quinoa Seeds

Following their harvest, the seed samples were oven-dried at 60 °C to ensure a constant weight to evaluate protein content according to the methods of [25]. The study assessed the nutritional composition of quinoa, focusing on protein content measured by the AOAC method, quantification of amino acids by HPLC, determination of minerals by atomic absorption, and analysis of vitamin C using the Ross method (1994). Additionally, the procedure for extracting phenolic compounds was detailed, including measurement of total phenolic content by the Folin–Ciocalteu method as described by [26], evaluation of ortho-diphenol content according to the method of [9], use of the aluminum chloride colorimetric method by [10] to identify total flavonoids, and finally, the determination of antioxidant activity by free radical scavenging tests (DPPH and ABTS) following the methods of [27].

##### Chemicals and Reagents

Chemicals such as Trolox, DPPH, ABTS, methanol, and other reagents were purchased from Sigma-Aldrich (Steinheim, Germany). Standards included catechin and various amino acids. All chemicals and reagents used were of analytical grade. Mineral and biochemical analyses were conducted during a 6-month internship at the CITAB research center, University of UTAD, Vila Real, Portugal.

## 5. Statistical Analysis

The dataset was analyzed by splitting the data by cropping year, and a one-way analysis of variance (ANOVA) was performed to assess the effects of treatments (T0, T1, T2) on the measured parameters. The significance level was set at *p* < 0.05. For post-harvest analyses of quinoa yield components, three samples per row for each treatment within each block were analyzed, resulting in a total of 27 samples per treatment (3 blocks × 3 rows × 3 samples per row). For chemical and biochemical analyses, a mixed sample from seedlings belonging to each treatment within each block was prepared, with analyses performed on three homogenized samples (one per treatment per block, n = 3).

Multivariate analyses, including Principal Component Analysis (PCA) and heatmaps, were performed using XLStat software (2024.3). These analyses were based on the Pearson correlation approach, computed from the mean values of all assessed parameters. PCA was employed to identify relationships and clustering patterns among treatments, while heatmaps provided a visual representation of the correlations between variables, offering deeper insights into the data structure. These multivariate approaches complemented the univariate analysis by highlighting trends and associations across the dataset.

## 6. Conclusions

This study conducted on white quinoa (*Chenopodium Quinoa* Willd.) under saline irrigation conditions in Cherfech provided significant results regarding the plant’s adaptability to these growing conditions. The three salinity treatments, represented by salt concentrations in irrigation water (T0, T1, and T2), induced marked variations in quinoa’s physiological and biochemical parameters. The most significant aspects of quinoa’s adaptability were its growth, seed yield, and biochemical responses under saline stress. Particularly, treatment T2, which had the highest salinity, demonstrated the plant’s remarkable capacity to maintain growth and seed yield, showcasing its resilience.

Biochemically, the significant increases in soluble proteins, nitrogen content, and bioactive compounds, such as vitamin C, phenols, flavonoids, and antioxidant activities (DPPH, ABTS), were crucial indicators of quinoa’s adaptability to saline conditions.

Treatment T2, with the highest salinity, led to maximal values in terms of growth, seed yield, and nutritional quality of quinoa. The content of soluble proteins and nitrogen showed significant increases from year to year, demonstrating quinoa’s ability to positively respond to salinity conditions. The most affected parameters were growth, protein content, and antioxidant activity. Treatment T2 showed the highest growth performance, including panicle height, biomass, and seed yield. Soluble protein levels and nitrogen content also increased significantly, suggesting enhanced metabolic activity under salt stress. Additionally, antioxidant compounds, including vitamin C, phenols, and flavonoids, were notably elevated in response to the saline irrigation.

Furthermore, bioactive compounds such as vitamin C, phenols, flavonoids, DPPH, and ABTS showed significant variations, with maximal values observed in treatment T2. Mineral element concentrations in the seeds also varied significantly between treatments, with T2 generally exhibiting the highest concentrations. These results emphasize the robustness and adaptability of white quinoa (*Chenopodium Quinoa* Willd.) to saline stress, offering promising prospects for its use in arid areas or those prone to high salinity conditions.

However, a negative trade-off was observed under T2 with a reduction in lysine levels, which might indicate metabolic reallocation. It is important to emphasize that the increase in other essential amino acids and bioactive compounds suggests a favorable trade-off toward stress adaptation. Regarding soil salinity, while T2 yielded optimal results for quinoa’s growth and nutritional quality, caution is needed for long-term salinity management. Prolonged exposure to high salinity levels may lead to soil degradation, affecting quinoa’s performance in the future. Therefore, it is essential to assess the sustainability of increased salinity over multiple growing seasons.

The increases in soluble proteins, bioactive compounds (such as vitamin C, phenols, and flavonoids), and antioxidants under saline stress are beneficial for both the plant’s stress response and human health. The elevated antioxidant levels suggest quinoa’s potential to offer enhanced nutritional value in saline environments, contributing to its classification as a superfood. These bioactive compounds not only help the plant withstand stress but also provide significant health benefits to humans, such as improved immunity and protection against oxidative stress.

These results are particularly applicable in regions facing salinity stress, such as coastal or arid areas with high salinity in irrigation water. Quinoa’s adaptability to saline conditions makes it a viable crop for regions where other crops struggle, contributing to food security and sustainable agricultural practices in these areas. It would be beneficial in farming systems that focus on drought-tolerant and salt-tolerant crops, offering a sustainable solution to soil salinity challenges.

The key mechanisms identified were enhanced antioxidant production, nitrogen uptake, and osmotic adjustments. Increased levels of antioxidants, such as vitamin C, phenols, and flavonoids, were critical to quinoa’s resilience under saline conditions, helping protect the plant from oxidative damage. Additionally, the increased nitrogen uptake in treatment T2 suggests improved nutrient absorption capabilities, likely due to better root development and osmotic adjustments, which are essential for the plant’s growth and stress tolerance.

This research enhances our understanding of how quinoa responds to environmental stress, paving the way for more sustainable farming practices in similar conditions. It underscores quinoa’s potential as a viable crop for cultivation in areas prone to salinity, providing both ecological and nutritional advantages, especially in arid and semi-arid climates. By addressing the challenges faced by vulnerable communities in these environments, the study offers a sustainable solution that promotes food security, supports socio-economic development, and contributes to rural growth.

One limitation of this study is that it primarily focused on quinoa’s physiological and biochemical responses under saline stress without evaluating the potential for genetic variability among different quinoa cultivars. Future research could investigate the adaptability of other quinoa varieties to saline conditions to identify those with superior performance. Additionally, the study did not consider long-term salinity impacts on soil health and productivity, which are critical for ensuring sustainable agricultural practices. Further investigations into soil management strategies to mitigate salinity effects are essential. Finally, a more detailed economic analysis of quinoa cultivation under saline conditions could provide valuable insights for farmers and policymakers aiming to implement quinoa as a staple crop in salinity-prone areas.

## Figures and Tables

**Table 1:** Initial state of soil physicochemical quality for the two years of experimentation.

Parameters	Initial State of the Soil in 2015	Initial State of the Soil in 2016
pH	7.9 ± 0.4	8.1 ± 0.3
EC (dS/m)	2.63 ± 0.3	2.98 ± 0.4
Ca^2+^ (me/L)	5.25 ± 0.6	5.25 ± 0.2
Mg^2+^ (me/L)	3.02 ± 0.4	3.22 ± 0.6
Na^+^ (me/L)	17.07 ± 0.5	18.34 ± 0.2
Cl^−^ (me/L)	15.19 ± 0.3	16.85 ± 0.6
N (mg/kg)	0.8 ± 0.1	0.8 ± 0.2
P (mg/kg)	16.2 ± 0.2	16.5 ± 0.2
K (mg/kg)	60.2 ± 1.3	63 ± 2.3

**Table 2:** Analysis of vitamin C content, phenols, flavonoids, and antioxidant activity of quinoa seeds grown in 2015 and 2016 with different saline water treatments.

Session	Treatments	Vitamin C (mg AA/100 g DW)	Total phenols (mg GAE/100 g DW)	Ortho-diphenols (mg GAE/100 g DW)	Flavonoids (mg CE /100 g DW)	ABTS (mg TEAC/100 g DW)	DPPH (mg TEAC/100 g DW)
**2015**	T0	6.59 ± 0.19 c	36.12 ± 0.65 c	6.08 ± 0.26 c	6.68 ± 0.07 c	20.210 ± 0.2 c	32.41 ± 0.5 c
	T1	7.04 ± 0.35 b	36.67 ± 0.68 b	7.79 ± 0.47 b	7.25 ± 0.11 b	27.710 ± 0.3 b	34.210 ± 0.9 b
	T2	8.02 ± 0.11 a	38.90 ± 0.35 a	8.09 ± 0.15 a	7.69 ± 0.07 a	31.410 ± 0.6 a	36.610 ± 0.9 a
**2016**	T0	7.63 ± 0.74 c	36.49 ± 0.60 c	7.40 ± 0.09 c	7.13 ± 0.12 c	22.2 ± 0.6 c	33.4 ± 0.1
	T1	8.57 ± 0.59 b	37.35 ± 0.73 b	9.11 ± 0.13 b	8.12 ± 0.07 b	28.3 ± 0.8 b	36.2 ± 0.1 b
	T2	9.88 ± 1.50 a	39.60 ± 0.45 a	11.76 ± 0.30 a	9.55 ± 0.17 a	33.2 ± 0.9 a	38.10 ± 0.5 a

Note: Means followed by different letters above the bars indicate significant differences (*p* < 0.05) between the different treatments: T0 (control, using tap water with EC = 1.4 dS/m), T1 (EC = 9.0 dS/m), and T2 (EC = 18 dS/m) according to the Tukey test.

**Table 3:** Mineral element concentration (mg/100 g DW) of quinoa seeds grown in 2015 and 2016 with different saline water treatments.

	First Growing Season 2015			Second Growing Season 2016		
Mineral Elements (mg/100 g DW)	T0	T1	T2	T0	T1	T2
K	888 ± 0.75 **b**	895 ± 0.91 **b**	900 ± 0.25 **a**	890 ± 0.34 **c**	900 ±0.19 **b**	910 ± 0.29 **a**
Na	10.2 ± 2.33 **b**	10.8 ± 3.18 **b**	11.2 ± 2.03 **a**	10.8 ± 1.39 **c**	11.2 ± 1.42 **b**	11.5 ± 3.27 **a**
P	251 ± 0.07 **c**	277 ± 0.10 **b**	300 ± 0.04 **a**	283 ± 0.02 **c**	334 ± 0.03 **b**	360 ± 0.08 **a**
Mg	200 ± 0.08 **c**	210 ± 0.09 **b**	215 ± 0.05 **a**	205 ± 0.04 **c**	222 ± 0.03 **b**	231 ± 0.05 **a**
Zn	4.00 ± 0.06 **c**	4.2 ± 0.04 **b**	4.4 ± 0.06 **a**	4.0 ± 0.01 **c**	4.2 ± 0.06 **b**	4.4 ± 0.04 **a**
Ca	140 ± 0.06 **c**	144 ± 0.32 **b**	146 ± 0.31 **a**	142 ±0.04 **c**	145 ± 0.04 **b**	148 ± 0.05 **a**
Fe	120 ± 0.07 **c**	127 ± 0.04 **b**	130 ± 0.08 **a**	122 ± 0.03 **c**	129 ± 0.06 **b**	131 ± 0.03 **a**

Note: Means followed by different letters above the bars indicate significant differences (*p* < 0.05) between the different treatments: T0 (control, using tap water with EC = 1.4 dS/m), T1 (EC = 9.0 dS/m), and T2 (EC = 18 dS/m) according to the Tukey test.

**Table 4:** Amino acid contents of quinoa seeds from two growing seasons irrigated with different saline water treatments.

		Seeds from the First Growing Season 2015			Seeds from the Second Growing Season 2015 2016		
	Amino Acid Concentrations (mg/g DW)	T0	T1	T2	T0	T1	T2
	Trp	30 ± 1.1 c	33 ± 1.0 b	35 ± 1.1 a	31 ± 1.0 c	34 ± 1.2 b	36 ± 0.9 a
	His	28.8 ± 1.3 c	29.8 ± 0.8 b	31.8 ± 1.0 a	29.8 ± 0.9 c	30.8 ± 0.5 b	32.8 ± 0.8 a
**Essential amino acid**	Thr	29.8 ± 1.1 c	30.8 ± 1.0 b	32.8 ± 0.9 a	33.8 ± 0.5 c	35.8 ± 0.7 b	36.8 ± 0.4 a
	Val	42.1 ± 0.9 c	44.1 ± 0.8 b	45.1 ± 0.7 a	43.1 ± 0.6 c	46.1 ± 0.6 b	47.1 ± 0.3 a
	Lys	56.2 ± 0.3 a	54.2 ± 0.4 b	53.2 ± 0.6 c	55.2 ± 0.6 a	53.0 ± 0.4 b	52.2 ± 0.7 c
	Leu	59.5 ± 0.4 c	61.5 ± 0.3 b	63.5 ± 0.5 a	62.5 ± 0.3 c	63.5 ± 0.3 b	64.5 ± 0.7 a
	Phe	42 ± 0.9 c	44 ± 0.8 b	48 ± 0.4 a	43 ± 0.8 c	45 ± 0.4 b	49 ± 0.5 a
	Tyr	18.9 ± 1.1 c	19.9 ± 1.2 b	20.9 ± 0.9 a	21.9 ± 0.4 c	22.9 ± 0.7 b	23.9 ± 0.1 a
**Non-essential** **amino acid**	Asp + Asn	80.3 ± 0.8 c	88.3 ± 0.4 b	90.3 ± 0.4 a	92.3 ± 0.6 c	93.3 ± 0.5 b	94.3 ± 0.5 a
	Ser	41.4 ± 1.2 c	41.6 ± 1.4 b	41.3 ±1.1 a	42.6 ± 2.4 c	43.00 ± 1.8 b	43.9 ± 1.7 a
	Glu + Gln	132.1 ± 0.8 c	134.1 ± 0.9 b	136.1 ± 0.4 a	133.1 ± 0.9 c	134.1 ± 0.4 b	136.1 ± 0.8 a
	Gly	49.2 ± 0.7 c	51.2 ± 0.4 b	52.2 ± 0.8 a	53.2 ± 0.4 c	55.2 ± 0.2 b	57.2 ± 0.4 a
	Arg	77.3 ± 0.5 c	78.3 ± 0.7 b	79.3 ± 0.9 a	78.3 ± 0.7 c	79.3 ± 0.8 b	80.3 ± 0.6 a
	Ala	42.6 ± 0.7 c	43.6 ± 0.9 b	44.6 ± 0.7 a	43.6 ± 1.1 c	44.6 ± 0.4 b	45.6 ± 0.7 a
	Tyr	18.9 ± 0.9 c	19.9 ± 0.4 b	20.9 ± 0.9 a	19.9 ± 0.9 c	20.2 ± 0.6 b	21.6 ± 0.9 a

Note: Means followed by different letters above the bars indicate significant differences (*p* < 0.05) between the different treatments: T0 (control, using tap water with EC = 1.4 dS/m), T1 (EC = 9.0 dS/m), and T2 (EC = 18 dS/m) according to the Tukey test.

**Table 5:** Physicochemical qualities of irrigation water.

		Average ± Standard Deviation	
Parameters	T0 (EC = 1.4 dS/m)	T1 (EC = 9.0 dS/m)	T2 (EC = 18 dS/m).
pH	7.1 ± 0.3	7.2 ± 0.3	7.5 ± 0.3
EC (dS/m)	1.4 ± 0.2	9.0 ± 0.2	18.0 ±0.2
HCO_3_^−^ (me/L)	4.5 ± 0.2	6.3 ± 0.2	7.6 ± 0.3
SO_4_^2−^ (me/L)	23.09 ± 0.2	25.7 ± 0.2	30.43 ± 0.3
Ca^2+^ (me/L)	6.8 ± 0.6	10.0 ± 0.6	16.0 ± 0.5
Mg^2+^ (me/L)	8.2 ± 0.2	13.5 ± 0.4	15.9 ± 0.1
Na^+^ (me/L)	6.6 ± 0.2	56.6 ± 0.2	96.6 ± 0.2
Cl^−^ (me/L)	9.3 ± 0.3	59.3 ± 0.3	99.3 ± 0.3
K^+^ (me/L)	2.2 ± 0.1	2.8 ± 0.1	3.2 ± 0.1

**Table 6:** Averages of selected climate parameters (2015–2016) from the Cherfech station (National Institute of Meteorology of Tunisia).

Avg. Max. Temp. (°C)	Avg. Min. Temp. (°C)	Avg. Rel. Humidity (%)	Rainfall (mm)	Max. Wind Speed (m/s)
	2015	2016	2015	2016
**March**	17.8	19.8	10	10
**April**	22.5	24.1	11.6	13.6
**May**	28.6	26.2	15.8	15.9
**June**	30.5	30.5	19.6	19.6

## Data Availability

Data will be made available on request.
